# Therapeutic activities of naringenin on efavirenz-induced sleep-like disorder in the midbrain of white albino mice

**DOI:** 10.22038/ijbms.2020.47043.10852

**Published:** 2020-11

**Authors:** Dosumu Olufunke, Akang Edidiong, Faniyan Oluwatomisin, Akanmu Alani

**Affiliations:** 1 Department of Anatomy, College of Medicine, University of Lagos, Idi-Araba, Lagos, Nigeria; 2 Department of Haematology and Blood Transfusion, College of Medicine, University of Lagos, Idi-Araba, Lagos, Nigeria

**Keywords:** cART, Dopamine, Efavirenz, Head twitch response, Naringenin, Oxidative stress

## Abstract

**Objective(s):**

Efavirenz, has proven to be effective in suppressing human immunodeficiency virus (HIV) viral load; however, complaints of sleep disorders including hallucination, and insomnia have greatly contributed to non-adherence to antiretroviral therapy. This study aimed at investigating therapeutic activities of naringenin on efavirenz-induced sleep disorder.

**Materials and Methods:**

Sixty mice were divided into six groups of control, combination antiretroviral therapy (cART), efavirenz, naringenin, naringenin/efavirenz and naringenin/cART. Efavirenz, cART, and naringenin were administered orally and daily at 15 mg/kg, 24 mg/kg and 50 mg/kg, respectively for 28 days. Post neurobehavioral test, oxidative stress, histology and immunohistochemistry for dopamine were carried out after administration process.

**Results:**

Efavirenz (*P<*0.0001) and cART (*P<*0.01) significantly increased immobility during open field (*P<*0.01), escape time in seconds (sec) in Morris water maze (*P<*0.001) and numbers of head-twitch response (HTR) (*P<*0.0001). Similarly, there was a significant increase in malondialdehyde (MDA) (*P<*0.0001) and decreased superoxide dismutase (SOD) (*P<*0.001) and reduced glutathione (GSH) (*P<*0.001); however, naringenin-treated groups potentiated anti-oxidant function by reducing oxidative stress (*P<*0.01). Histological evaluation demonstrated severe neurodegeneration, vacuolization and pyknosis in efavirenz and cART compared to naringenin groups. Dopaminergic neurons using immunohistochemial antibody (tyrosine hydroxylase) staining showed poor immunoreactivity in efavirenz and cART in contrast to naringenin groups.

**Conclusion:**

Efavirenz and cART have the potential of inducing sleep disorder possibly due to their capability to trigger inflammation and deplete dopamine level. However, naringenin has proven to be effective in ameliorating these damages.

## Introduction

Human immunodeficiency virus infection and acquired immune deficiency syndrome (HIV/AIDS) has been one of the world`s leading epidemic. In spite of frantic efforts made by the World Health Organization (WHO) and other civil services, the epidemic remains daunting with a staggering number of about 36.7 million persons living with HIV/AIDS (PLWHA). From this burden, it has been estimated that about 1.9 million PLWHA are found in Nigeria (1). The management of this disease has been fostered by the efficacy and effectiveness of combination antiretroviral therapy (cART) comprising non-nucleotide reverse transcriptase inhibitors, nucleos(t)ide reverse transcriptase inhibitors, protease inhibitors, fusion (entry) inhibitors and integrase strand transfer inhibitors (1, 2). This therapy has remarkably reduced the mortality and morbidity rate associated with HIV (3), warranting the WHO ambitious target of sustained antiretroviral therapy for at least 90% of people diagnosed with HIV infection by 2020 and 95:95:95 for treatment target by 2030 (1, 4, 5).

Efavirenz is a selective inhibitor of non-nucleoside analogue and non-competitive inhibitor of the reverse transcriptase of HIV. It attaches directly to the enzyme and blocks the activities of DNA–RNA polymerase, causing the destruction of the catalytic location of the enzyme (4). Efavirenz has been effective in suppressing viral load; however, it has led to various hypersensitivity, hepatotoxicity, and neuropsychiatric toxicity effects, therefore increasing concerns about adherence to therapy (6-9). Sleep disorders have been a major side effect of efavirenz therapy (both as a monotherapy and combination therapy) (7, 10, 11). About 26% of people living with HIV subjected to efavirenz therapy have been reported to suffer from sleep disorders of various forms including somnolence, insomnia and hallucination (7, 9)

Bearing in mind the side effects of efavirenz, there is therefore an urgent need for an adjuvant therapy to promote Antiretroviral (ARV) adherence, longevity of life, and minimizing the spread of the virus. Hence, naringenin a bioflavonoid abundant in citrus fruits especially grapes (12) was used as an adjuvant therapy in the present study. Head twitch response (HTR), a neurobehavioral test in animal model, has been reported as a proxy to hallucination in human (10, 11, 13). Therefore, we evaluated the hallucination (sleep disorder) activities of efavirenz via the HTR neurobehavioral test. Furthermore, naringenin has been reported in our previous studies to effectively combat cART-induced cerebellar disorder and memory deficits (12, 14) as well as testicular disorders and sperm DNA fragmentation (15, 16). 

Therefore, this study aimed at investigating and validating the hallucinogenic property of efavirenz and a possible means of ameliorating this side effect of efavirenz via concomitant administration with naringenin, a bioflavonoid.

## Materials and Methods

***Animals***

Sixty adult male white albino mice weighing 25±5 g procured from Animal laboratory centre of College of Medicine, University of Lagos were used for this experiment. They were housed in standard animal house of the Department of Anatomy, College of Medicine of the University of Lagos and exposed to 12 hr/12 hr light/dark cycle. All experimental procedures were conducted between 8:00 and 11:00 hr and were carried out in accordance with the standard international guidelines on the use of animals for research. Approval for the study was obtained from the Health Research Ethics Committee on Animals Use, College of Medicine, University of Lagos, Nigeria (CMUL/HREC/10/18/442).

***Chemicals and drugs***

Efavirenz (600 mg) and cART (EFV+3TC+TDF) in tablet form were obtained from the Aids Prevention Initiative of Nigeria (APIN) Clinic, Lagos University Teaching Hospital. Naringenin was ordered from Sigma Aldrich, South Africa. Efavirenz, cART and naringenin were dissolved in distilled water. The drugs were prepared daily for the study.

***Experimental design***

Mice were randomly allotted into 6 groups (control- CNTL, naringenin-nan, cART, efavirenz-efv, naringenin/cART and naringenin/efavirenz) (n=10). Efavirenz (15 mg/kg) (10, 15), naringenin (50 mg/kg) (17) and cART (24 mg/kg) (18) were dissolved in distilled water and administered orally, daily for 28 days. The control received distilled water. The weight of the animals were monitored daily before feeding and administration processes. Neurobehavioral tests (open field and Morris water maze) were conducted before the commencement and after administration. HTR was conducted 30 min after administration. Neurobehavioral test operations were observed and scored independently by two experienced blind observers. 

***Neurobehavioral evaluation***

*Head twitch response*

HTR, a proxy of hallucination in humans that is a violent rapid lateral movement of the head similar to pinna reflex (10) was carried out using a cage similar to the animal’s cage with a digital video camera attached according to the method described by Gatch *et al.,* (2013) with slight modification in the time taken to observe the animals’ response activities (10). Quantification of the response was carried out according to the grading method previously described where the scoring was performed by blind observers and analysed using analysis of variance (ANOVA) (10, 13).

*Morris water maze (MWM)*

A round pool of water about 6 feet in diameter and 3 feet deep constituted the major component of the water maze. A video camera that covered all sides of the maze was used for recording the task. Initially, the water maze tank was filled with tap water at temperature of 26°C and the platform placed at the south-west location (SW) of the water tank. During training, the platform was exposed 1 cm above the water level. After training, the water surface was made opaque and the platform was submerged 1 cm below the surface of the water. During the experiment (pre and post test), the animals were dropped into the water at different starting points (north, south, west and east) with the platform stationed at the same location as in the training test that lasted for 3 days. The animals were monitored and the time taken to locate the platform was recorded (19).

*Open field test*

Locomotor activities were evaluated in an open field area, which consisted of a square wooden box (40 x 40 cm, walls 40 cm high) divided into 16 sub squares (10, 11). The test was initiated by placing the mice at the centre of the arena. The behaviour of the mice was then observed for 5 min. After each test, the arena was thoroughly cleaned with cotton pad wetted with 70% ethanol. The parameters analysed during the test were number of line crossings (crossing the square boundaries with both forepaws), rearing (standing on its hind legs), grooming (rubbing the body with paws or mouth and rubbing the head with paws) and duration of immobility (10, 11)

***Oxidative stress marker assay***

Mice were deeply anaesthetized intraperitoneally (ketamin- 100 mg/kg and xylazine- 4 mg/kg), the midbrain was quickly harvested, weighed and fixated on ice for oxidative stress markers (SOD, catalase- CAT, GSH and MDA) assay. Specimens were later homogenized in cold PBS (pH 7.4) with Teﬂon tissue Homogenizer. The homogenates were centrifuged at 14,000 rpm for 15 min at 4 ^º^C. The supernatant was used to measure the tissue malondialdehyde (MDA), superoxide dismutase (SOD), catalase (CAT) and reduced glutathione (GSH) activity levels. Total SOD activity in tissue homogenates was determined based on the ability of SOD to inhibit the autoxidation of pyrogallol. CAT was assayed calorimetrically at 620 nm and expressed as moles of hydrogen peroxide (H_2_O_2_) consumed/min/mg protein (20, 21). GSH was determined by the method described by Ellman (22). Trichloroacetic acid (TCA) 10% (equal volume) was added to the homogenate and centrifuged (23). The assay for membrane lipid peroxidation (LPO) was performed in accordance with some modifications from Tsikas (23). The amount of MDA formed in each of the samples was assessed by measuring the optical density of the supernatant at 532 nm (22, 23).

***Histological and immunohistochemical studies***

*Histological procedure*

Mice were anaesthetized intraperitoneally (ketamine- 100 mg/kg and xylazine- 4 mg/kg) and perfused transcardially with 0.1 M PBS (pH 7.4) and 10% formalin in 0.1 M PBS (pH 7.4). The midbrain tissues were immediately harvested and fixated in 10% buffered formalin. The tissues were then dehydrated, embedded in paraffin and coronal sections (5 µm) were obtained with rotatory microtome. Each section was stained with haematoxylin and eosin (H&E) dyes for basic histological demonstration (24). 

*Immunohistochemistry protocol for Glial fibrillary acidic protein (GFAP) and dopamine*

Astrocytes were demonstrated by immunohistochemical staining for GFAP, and dopaminergic cells were demonstrated by immunohistochemical staining with primary (tyrosine hydroxylase) and secondary antibodies (mouse specific) for the expression of dopamine. Antibodies were procured from Novocastra (GFAP- GA5- dilution ratio 1: 100, tyrosine hydroxylase - TH, 1B5- dilution ratio 1:50). All protocols were carried out according to guidelines by immunohistochemistry world (IHC) staining descriptions (25). For dopaminergic cell and GFAP expression, blocking and antibody incubations were performed with a solution of 1% BSA and 0.1% Triton X-100 (Sigma-Aldrich, Singapore) in PBS. The samples were blocked for 1 hour at room temperature, incubated overnight in respective primary antibody solutions at 4 ^°^C, and incubated for 2 hr in secondary antibody solutions at room temperature. After the primary and secondary antibody incubations, the sections were washed four times in PBS and incubated in DAB for 5 min, washed briefly, counterstained in haematoxylin, dehydrated in graded ethanol and cleared in xylene (26).

***Statistical analysis***

Data from results of neurobehavioral tests and oxidative stress markers were expressed as the means±SEM (standard errors of the mean) and analyzed with one-way (for single variable grouped data comparison) and two-way (for multiple variable grouped data comparison) analysis of variance (ANOVA) as appropriate followed by a Tukey’s test. The significance level of the analyses was set at *P*<0.05. Statistical program used was GraphPad Prism 7.0 Version for Windows, GraphPad Software (San Diego, CA, USA).

## Results

***Neurobehavioral test result***

*Head twitch response analysis*

**Figure 1:**
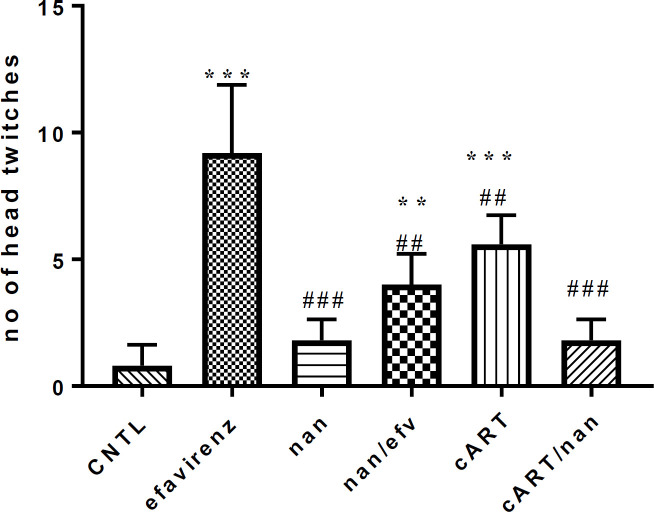
Head twitch response comparison among experimental group of mice

^*^=*P<*0.05, ^**^=*P<*0.01, ^***^=*P<*0.001 compared to the control; ^#^=*P<*0.05, ^##^=*P<*0.01, ^###^=*P<*0.001 between treatment groups and efavirenz group CNTL: control, nan: naringenin, efv: efavirenz, cART: combination antiretroviral therapy

Results showed a statistically significant increase in HTR in the efavirenz, efavirenz/naringenin and cART groups compared to control, (*P*<0.001). However, there was a statistically significant decrease in HTR in cART/naringenin compared to cART, and efv/naringenin compared to cART, and efv/naringenin compared to efavirenz only treated group (P<0.01) (Figure 1).

*MWM assessment*

**Figure 2:**
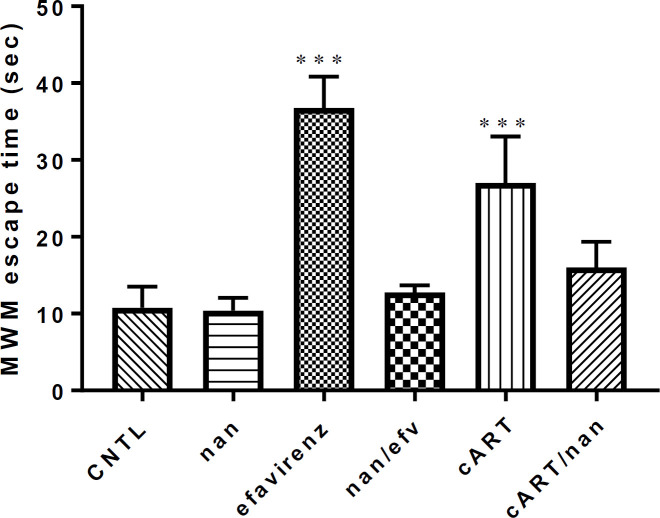
Morris water maze test escape time in second comparison among experimental group of mice

^ ***^=*P<*0.001 compared to the control

There was a statistically significant increase (*P*<0.0001) in time taken for animals in the efavirenz and cART-treated groups to locate the hidden platform compared to the control group. There was no statistically significant difference in the time (sec) taken for animals in efavirenz /naringenin, naringenin, and cART/naringenin-treated groups to locate the hidden spot during the test compared to those in the control group (Figure 2).

*Open field test assessment*

**Figure 3:**
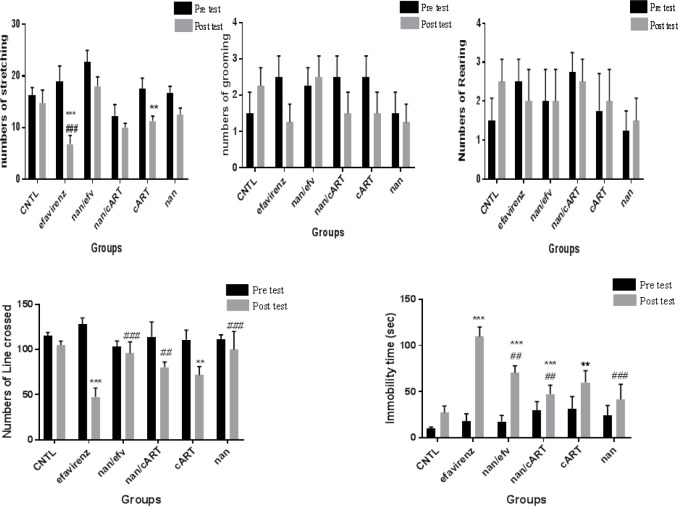
Open field assessment

^*^=*P<*0.05, ^**^=*P<*0.01, ^***^=*P<*0.001 significant differences between pre test and post test of each group; ^#^=*P<*0.05, ^##^=*P<*0.01, ^###^=*P<*0.001 significant differences between post test of treatment groups and control group

In the numbers of rearing and grooming, there were no statistically significant differences between pre and post test of each group. Post test comparison among the groups showed no statistically significant differences. There was a statistically significant decrease in line crossing activities between pre and post test in efavirenz (*P*<0.0001) and cART (*P*<0.001)0treated groups. However, groups treated with naringenin both as an adjuvant and monotherapy showed a statistically significant increase (*P*<0.01) in post test assessment for line crossing compared to efavirenz-treated group. The pre and post test assessment in groups treated with efavirenz and cART showed significant increase (*P*< 0.01) in immobility time (sec), while groups treated with naringenin (Efv/naringenin and cART/naringenin) had significant reduction (*P*< 0.01) in immobility time (Figure 3).

***Oxidative stress markers (SOD, CAT, GSH and MDA) of the midbrain in efavirenz, cART and naringenin treatment***

**Figure 4:**
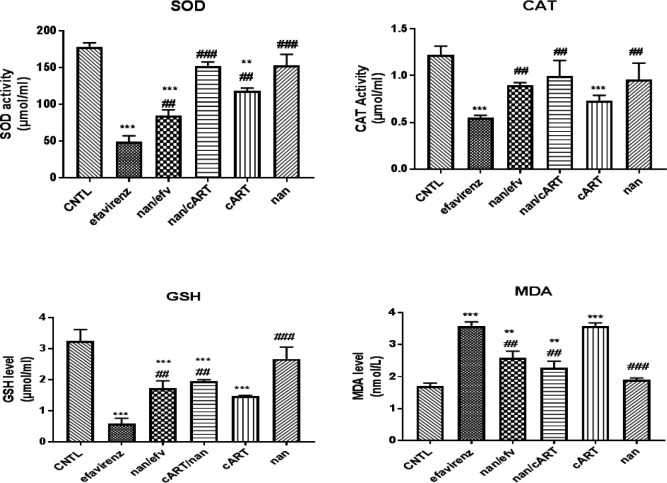
Assessment of Oxidative Stress Markers

^*^=*P<*0.05, ^**^=*P<*0.01, ^***^=*P<*0.001 compared to control group; ^#^=*P<*0.05, ^##^=*P<*0.01, ^###^=*P<*0.001 compared to efavirenz group

Naringenin-treated group showed no statistically significant difference in SOD, GSH and CAT activity levels when compared to control group. There was a statistically significant decrease in SOD (*P*<0.0001), CAT (*P*<0.001) and GSH (*P*<0.0001) levels in efavirenz and cART-treated groups compared to the control group. However, there was a statistically significant increase in SOD (*P*<0.001), CAT (*P*<0.01) and GSH (*P*<0.01) level in naringenin/efavirenz and naringenin/cART-treated groups compared to efavirenz-treated groups (Figure 4). 

Analysis of MDA level elucidated that there was a statistically significant increase (*P*<0.0001) in MDA level in efavirenz and cART-treated groups, while naringenin-treated groups showed no significant changes compared to the control group (Figure 4). Naringenin/efavirenz and naringenin/cART-treated groups, however, showed a statistically significant decrease (*P*<0.001) in MDA level compared to efavirenz-treated group (Figure 4).

***Histology assessment of the midbrain in efavirenz, cART and naringenin treatment***

**Figure 5:**
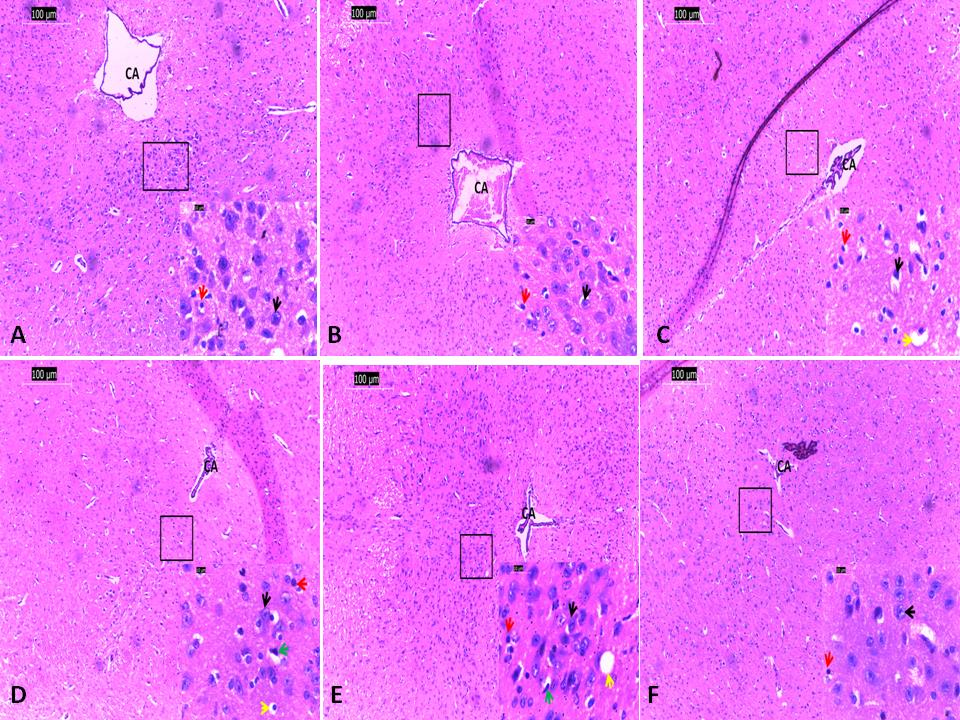
Basic histology demonstration of nuclei of periaqueductal gray area of the midbrain of both control and experimental groups. (H&E x100 (scale: 100 ųm) and x1000 (scale: 10ųm) (A: Control group, B: Naringenin group, C: combination antiretroviral therapy (cART) group, D: cART/Naringenin group, E: Efavirenz group, F: Efavirenz/Naringenin groupCA: cerebral aqueduct, Black arrow: neuronal cell body, Red arrow: neuroglial, Green arrow: Pyknosis, Yellow arrow: intracytoplasmic vacuolization)

Cyto-architecture of the periaqueductal gray area of the midbrain tissue of the control and naringenin-treated groups appeared normal with the presence of properly aligned choroid plexus within the cerebral aqueduct. Nuclei of neuronal cell bodies appeared fusiform shaped, centrally placed and basophilic. The cytoplasm appeared eosinophilic (Figure 5). Varying degree of cellular alterations including pyknosis of the nuclei (intensely darkly stained), intracytoplasmic vacuolisation, influx of neuroglial cells and constriction of the chroid plexus (degeneration of the ependyma cells) of the cerebral aqueduct were observed in mice treated with efavirenz, cART, efavirenz/naringenin and cART/naringenin with varying intensities. These pathological alterations were highly exhibited in efavirenz and cART-treated groups, while efavirenz/naringenin and cART/naringenin-treated groups exhibited reduced evidence of these pathological changes (Figure 5).

***Immunohistochemistry assessment GFAP in the midbrain of efavirenz, cART and naringenin treatment***

**Figure 6:**
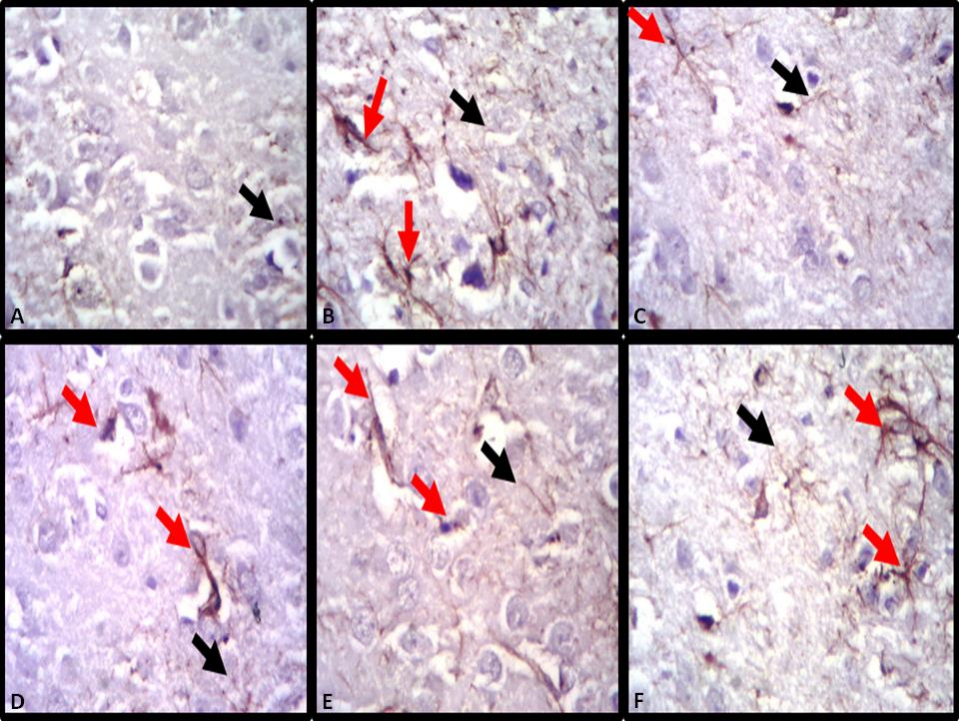
Glial fibrillary acidic protein expression for astrocytic demonstration in midbrain of control and experimental groups (A: Control group, B: Naringenin group, C: combination antiretroviral therapy (cART) group, D: cART/Naringenin group, E: Efavirenz group, F: Efavirenz/Naringenin group, red arrow: activated astrocytes, black arrow: deactivated astrocytes)

Mice in the control group exhibited no signs of activated and hypertrophied astrocyte. Combined expressions of severely hypertrophied and poorly activated astrocytes were demonstrated in efavirenz and cART-treated groups. Hypertrophied astrocyte showed long protoplasmic processes and robust cell body, and activated astrocytes showed pronounced expression for GFAP. Naringenin, efavirenz/naringenin and cART/naringenin-treated groups showed mild hypertrophied with numerous activated astrocytes (Figure 6).

***Immunohistochemistry assessment of dopamine in the midbrain of efavirenz, cART and naringenin treatment***

**Figure 7:**
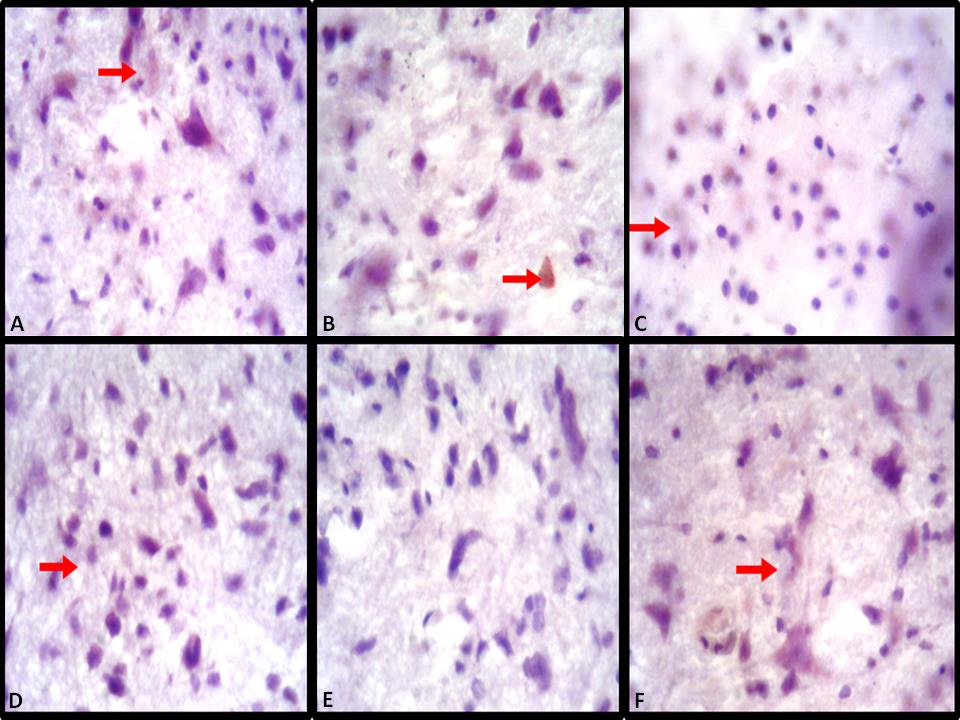
Dopamine antibody reactivity for dopamine demonstration in midbrain of experimental groups. (A: Control group, B: Naringenin group, C: combination antiretroviral therapy (cART) group, D: cART/Naringenin group, E: Efavirenz group, F: Efavirenz/Naringenin group, red arrow: immunopositivity)

Dopaminergic neurons of the control group exhibited positive immunoreactivity to dopamine immunohistochemical antibody. However, efavirenz and cART-treated groups showed the relatively poor immunoreactivity expression to dopamine antibody. On the contrary, naringenin-treated groups showed improved immunoreactivity for dopamine antibody used to demonstrate the level of dopamine expression (Figure 7). 

## Discussion

Efavirenz, a Non-Nucleotide reverse transcriptase inhibitor (NNRTI), has been proven to be highly effective in suppressing HIV viral load in PLWHA (27) and also a psychoactive drug for people using it for recreational purposes (28, 29). The use of efavirenz for these purposes has brought about various neuropsychiatry effects including depression, anxiety, and sleep disorders (hallucination, abnormal dreaming and insomnia). This is a major complain by people subjected to the drug and has resulted in the non-adherence to the therapy. Hence, it is in the process of being withdrawn from firstline therapy for PLWHA despite its effectiveness in suppressing HIV viral load (30, 31, 32).

Various pharmacological mechanisms have been suggested to be ways by which efavirenz evokes its neuropsychiatric effect, but unfortunately little has been suggested as effective means of ameliorating these effects (10, 11, 33, 34). 

The HTR, a proxy to hallucination in humans, has been discussed to be initiated in animal models by hallucinogenic drugs such as Lysergic acid diethylamide and 2,5-Dimethoxy-4-iodoamphetamine, which are serotonergic receptor- hallucinogenic receptor regulator (5HT2a) partial agonist. HTR analysis of this study indicates that the use of efavirenz and cART have the potential of stimulating hallucination thereby affecting sleep quality in people living with HIV subjected to the therapies (9, 11).

The induction of significant HTR in efavirenz and cART-treated groups may be due to their ability to stimulate 5HT2a receptor of the periaqueductal gray of the midbrain (10, 15). From the study, cART-treated group showed a decrease in HTR compared to the efavirenz-treated group. This could be as a result of competition by other pharmacological substances of the combination therapy leading to a decrease in the concentration of efavirenz needed to activate 5HT2a receptor. This result is consistent with findings by Gatch *et al.,* (2013) who reported that efavirenz induced HTR in mice similar to Lysergic acid diethylamide (LSD) and other related hallucinogens via the activation of 5-HT2A receptor and also a decrease in efavirenz affinity for the receptor resulted in a decrease in the number of HTR observed (10). Reduction in HTR in groups treated with naringenin as compared to cART and efavirenz-treated groups maybe as result of therapeutic activities of naringenin on the serotonergic system via competing as 5HT2a receptor antagonist thereby reducing the concentration needed to activate the receptor. This correlates with the findings that naringenin exerts antidepressant effect via activities on serotonergic and noradrenergic systems (35). 

 Serotonergic and dopaminergic system have been proven to be involved in motor coordination (10, 11, 33). Significant decrease in motor activities of animals in efavirenz and cART-treated groups maybe associated with neurodegenerative changes in the nuclei located in the periaqueductal gray area of the midbrain, which have been associated with motor coordination functions (10). This is in agreement with Cavalcante *et al.,* who reported decrease in exploratory activities in open field in his study on HIV antiretroviral drug efavirenz and induction of anxiety-like and depression-like behaviors in rats at both acute and subchronic administration doses. He attributed the decrease in exploratory ability of the rats to marked alterations in the concentrations of monoamines and their metabolites in the brain (11). 

Quality of sleep has been associated with neuroplasticity activities of the brain in memory function (36, 37). Cognitive deficit associated with sleep deprivation has been suggested to be as a result of elevation in oxidative stress (36). The recticular activating system comprising of neuronal communication between the midbrain and different areas of the cerebral cortex including the hippocampus, limbic system, thalamus and the prefrontal context have been linked with interwoven brain functions such as cognition, alertness and sleep regulation (38). Memory consolidation has been linked to sleep quality (39). MWM assessment of this study infers that sleep deprivation effect of efavirenz and cART might have directly or indirectly impaired cognitive function through damage or alteration of recticular activating system of the brain involved with both the cognitive function and sleep quality (38). Anti-oxidant functions of naringenin in this study have helped to boost cognitive function in the group that received it as an adjuvant. These findings correlate with the study by Akang *et al.,* (2019) who reported that animals that received both cART and bioflavonoids (Naringenin and quercitin) had marked improvement in cognitive function of the hippocampus compared to cART-treated group (14). 

Oxidative stress, an imbalance in the production of free radicals resulting in inability of the body to detoxify harmful effects through neutralization by anti-oxidant, have been documented to be a possible mechanism for neurodegenerative disorders (15, 40, 41). The use of anti-oxidants such as naringenin and quercetin as adjuvant has been proven to be effective in ameliorating some of these effects (42, 43). Oxidative stress findings of this study insinuate that efavirenz and cART evoke their neuropsychiatric effects by their potency to reduce SOD, GSH, and CAT and increase the MDA. This result is similar to findings by Oremosu *et al.,* (2018) that cART increases MDA levels and also decreases CAT and SOD levels. Decrease in oxidative stress through increase in anti-oxidant activities displayed in groups treated with naringenin alongside cART and efavirenz was as a result of therapeutic anti-oxidant activities of naringenin. This is in correlation with various studies that used naringenin as an anti-oxidant adjuvant for combating increased oxidative stress posed by neurodegenerative disorders (18, 42, 44).

Efavirenz and cART-treated groups in the present study showed abundance of vacuolisation, pyknosis and neurodegeneration in the cytoarchitecture of the periaqueductal gray area of the midbrain of these groups indicating that efavirenz and cART have the potential of initiating neurodegeneration within the midbrain. This agrees with the findings of earlier reported studies that efavirenz triggered neurodegeneration of cells of the inferior colliculi (45) and cART initiated neurodegeneration in the Purkinje cells of the cerebellum (18). Reduction in pathological changes observed in naringenin/efavirenz and naringenin/cART insinuate recovery and improvement in neuronal cyto-architecture. This indicates that naringenin, which was administered as an adjuvant, maybe an effective anti-oxidant in reducing oxidative stress and ameliorating neuronal damage. This finding agrees with other studies that naringenin has therapeutic benefits by potentiating the activities of anti-oxidant enzymes. Hence, preventing the onset/deleterious impact of reactive oxygen species on cellular morphology (18) can lead to excellent improvement in neuronal damage (46).

Activation of astrocytes in neuronal damage has been documented to reduce the spread and persistence of inflammatory cells (47). Also, activated astrocytes during inflammation secrete neurotrophins (BDNF, TGF) to enhance neuronal survival as well as secretion of anti-oxidant such as glutathione to reduce oxidative stress (48). In the present study, the presence of astrocytic hypertrophy (excessive absorption of water due to alterations of aquaporin-4) and poor reactivity (due to loss of GFAP intermediate filament) in cART and efavirenz-treated groups indicated that the two substances have the potential to initiate cellular inflammation, impairment of the function of astrocytes in repairing as well as secreting anti-oxidants to reduce oxidative stress as a result of inflammation (49, 50). Improved reactivity of astrocytes to GFAP antibody demonstrated in the naringenin-treated groups suggests the potency of naringenin to stimulate astrocytic functions (anti-oxidant and neurotrophin secretion). De Sampaio *et al.,* (2010) demonstrated that secretion of neurotrophic factor, by astrocytes to encourage cellular repair, survival and growth, was increased by the administration of naringenin. Similarly, improved reactivity of astrocytes demonstrated in naringenin/efavirenz and naringenin/cART-treated groups maybe as a result of naringenin to increase GFAP level in astrocytes (49, 51). Dopaminegic neurons of the mid brain and it receptors at different part of the central nervous system including the hippocampus and limbic system have been reported to play a crucial role in mediating sleep, reward and learning function (10, 52). Similarly, patients with neurodegenerative disorders associated with deficit of dopamine neurons (of the substantial nigra and periaqueductal gray area) such as Parkinson’s syndrome have been reported to have sleep disorders of varying forms, especially daytime sleepiness (53, 54). In this study, efavirenz and cART decreased immunoreactivity to dopamine antibody. This implies that efavirenz and cART may have interacted with dopaminergic neurons of the midbrain, encouraging excessive release of dopamine, leading to reduction in sleepiness. This finding agrees with the work of Lim and colleagues who reported that sleep-deprived mice showed a significant decrease in dopamine receptor (54). In addition, Cavalcante *et al.,* (2017) reported a significant decrease in striatal dopamine level in sub-chronic efavirenz-treated groups compared to acute ones. Similarly, poor compensation for energy needed to balance excessive release of dopamine by dopaminergic cells of the midbrain have been attributed to be a possible cause of neurodegeneration in Parkinson’s disease (55). This might have resulted in the various phases of neurodegeneration observed in the histological assessment of this study.

Naringenin, a bioflavonoid, has been used in several studies to ameliorate depletion of dopamine level in Parkinson’s disease as a result of damage to dopaminergic neurons (25, 35, 56). Zbarsky *et al.,* related neuroprotective function of naringenin in the 6-OHDA model of Parkinson’s disease to its *anti-oxidant* capability and the ability to penetrate through the blood brain barrier into the brain. Hence, from the present study, naringenin-treated group demonstrated improved immunopositivity for dopamine markers. This suggests that naringenin enhanced the release of dopamine by reducing oxidative stress caused by efavirenz and cART on dopaminergic neurons, thereby improving their functions (56, 57). 

CNTL: control, nan: naringenin, efv: efavirenz, cART: combination antiretroviral therapy

CNTL: control, nan: naringenin, efv: efavirenz, cART: combination antiretroviral therapy

CNTL: control, nan: naringenin, efv: efavirenz, cART: combination antiretroviral therapy, SOD: Superoxide dismutase, GSH: Reduced Glutathione, CAT: Catalase, MDA: Malondialdehyde

## Conclusion

Finding of this study with respect to other studies suggests that efavirenz and cART containing efavirenz induce sleep disorder through their neurodegenerative actions on nuclei (including the dopaminergic neurons) of the periaqueductal gray areas of the midbrain. On the other hand, naringenin potentiates its therapeutic functions by its ability to cross the blood brain barrier as well as its ability to reduce oxidative stress, improve dopamine, GFAP reactivity and neuronal cell body of the periaqueductal area of the midbrain. 
